# Neurological complications of varicella zoster virus reactivation: Prognosis, diagnosis, and treatment of 72 patients with positive PCR in the cerebrospinal fluid

**DOI:** 10.1002/brb3.2455

**Published:** 2022-01-18

**Authors:** Tiphaine Lenfant, Anne‐Sophie L'Honneur, Brigitte Ranque, Benoit Pilmis, Caroline Charlier, Mathieu Zuber, Jacques Pouchot, Flore Rozenberg, Adrien Michon

**Affiliations:** ^1^ Université de Paris, Service de Médecine Interne Hôpital Européen Georges Pompidou, AP‐HP Paris France; ^2^ Université de Paris, Service de Virologie Hôpital Cochin, AP‐HP Paris France; ^3^ Équipe Mobile de Microbiologie Clinique Groupe Hospitalier Paris Saint Joseph Paris France; ^4^ Université de Paris, Equipe Mobile Infectiologie Hôpital Cochin Port‐Royal, AP‐HP Unité Biologie des Infections, Institut Pasteur, Inserm U1117 Paris France; ^5^ Service de Neurologie et Neurovasculaire Groupe Hospitalier Paris Saint Joseph Paris France

**Keywords:** cerebrospinal fluid, meningitis, neurological complications, prognosis, varicella‐zoster virus

## Abstract

**Background:**

VZV infection can involve every level of the neurologic system: from the central nervous system (CNS) to the peripheral nervous system (PNS), including aseptic meningitis. Prognosis seems to differ between these neurological involvements. Prognostic factors remain unknown.

**Methods:**

This is a retrospective multicenter study including all patients with a positive VZV polymerase chain reaction (PCR) in the cerebrospinal fluid (CSF) from eight centers in Paris (France) between 2011 and 2018. Unfavorable outcome was defined as mortality linked to VZV or incomplete recovery. Modified Rankin Scale (mRS) evaluated disability before and after the infection, with the difference designated as Rankin Delta.

**Results:**

Seventy‐two patients were included (53% male, median age 51 years, median mRS 0). Immunosuppression was reported in 42%. The clinical spectrum included 26 cases of meningitis, 27 instances of CNS involvement, 16 of PNS involvement, and 3 isolated replications (positive PCR but no criteria for neurological complications from VZV). Antiviral treatment was administered to 69 patients (96%). Sixty‐two patients completed follow‐up. Death linked to VZV occurred in eight cases. Unfavorable outcome (UO) occurred in 60% and was significantly associated with a higher prior mRS (Odd‐ratio (OR) 3.1 [1.4–8.8] *p* = .012) and the presence of PNS or CNS manifestations (OR 22 [4–181] *p* = .001, OR 6.2 [1.3–33] *p* = .03, respectively, compared to meningitis). In the CSF, higher protein level (*p* < .0001) was also significantly associated with a higher Rankin Delta.

**Conclusions:**

Neurological complications of VZV with evidence of CSF viral replication are heterogeneous: aseptic meningitis has a good prognosis, whereas presence of CNS and PNS involvement is associated with a higher risk of mortality and of sequelae, respectively.

## Graphical abstract

Neurological complications of varicella zoster virus reactivation can involve the central and peripheral nervous systems. Central nervous system involvement was associated with a higher mortality and peripheral involvement with higher sequelae. Higher age and Rankin scale prior to infection were associated with a poorer prognosis.

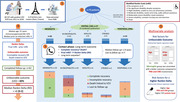

## 1 INTRODUCTION

Varicella zoster virus (VZV) is a double stranded DNA virus from the Herpesviridae family (Davison & Scott, 1986). After primary infection, the virus establishes latency principally in vertebral and cranial sensory ganglia which are the sources of reactivation as cellular immunity decreases (Cohrs & Gilden, 2007; Levin et al., 2003). VZV reactivation may be complicated by either local, neurological, or disseminated involvement. Among many identified risk factors, age and cellular immunodepression have been identified as the most important for VZV reactivation and complications (Kawai et al., 2014; Schmader, 2001).

Neurological complications from VZV are of primary concern. The virus can cause peripheral nerve involvement (cranial nerve including Ramsay‐Hunt syndrome, radiculopathy) and central neurological involvement (meningitis, meningoencephalitis, myelitis, stroke, VZV vasculopathy, necrotizing retinitis) (Gilden et al., 2015; Herlin et al., 2021; Nagel & Gilden, 2014; Steiner & Benninger, 2018). VZV vasculopathy, classically present as an ophthalmic zoster followed by acute contralateral hemiplegia or as headache, mental status changes, or focal deficit, may be underestimated in clinical practice because deficit may appear up to one year after herpes zoster (HZ) (Gilden et al., 2009; Sreenivasan et al., 2013). Several studies have reported a potential role for VZV in neurocognitive impairment (Chen et al., 2018; Itzhaki, 2018; Tsai et al., 2017) or giant cell arteritis (Gilden et al., 2015). Neurological manifestations can precede or even present without skin lesions of HZ as in zoster sine herpete (Blumenthal et al., 2011; Gilden et al., 2010). Involvement of the central nervous system (CNS), and especially myelitis or cerebral vasculitis, were described as associated with immunosuppression, but data are controversial as it has not been shown by more recent studies (Choi et al., 2014; Corral et al., 2020; Gilden et al., 1994; Nagel et al., 2008). Only a few studies have specifically addressed VZV meningitis, as cases are often reported among other causes of viral meningitis, or among the large dataset of all VZV neurologic complications (Aberle et al., 2005; Corral et al., 2020; Gilden et al., 2015; Nagel & Gilden, 2014). Follow‐up data are scarce to provide insights on potential neurologic and general sequelae of the infection (Becerra et al., 2013; Frantzidou et al., 2008; Grahn & Studahl, 2015; Kaewpoowat et al., 2016; Nowak et al., 2003; Persson et al., 2009; Pollak et al., 2012). Our hypothesis was that VZV aseptic meningitis, when not associated with other neurological complications, had a favorable prognosis compared to central or peripheral neurologic involvements. Our objectives were to describe the clinical and biological presentation, the therapeutic management (especially of the aseptic meningitis for which it is not codified), the morbimortality of VZV neurological complications, confirmed by positive VZV polymerase chain reaction (PCR) in the cerebrospinal fluid (CSF), and to look for prognostic risk factors. To fulfill these objectives, we conducted a multicenter retrospective study with one follow‐up update assessing the neurologic and general outcomes of patients with VZV neurological complications.

## 2 PATIENTS AND METHODS

### 2.1 Study design

We conducted a retrospective multicenter observational study that involved 20 medical departments from four French tertiary hospitals: Hôpital Necker‐Enfants Malades, Hôpitaux Universitaires Paris Centre (Cochin, Broca, Hôtel‐Dieu), Hôpitaux Universitaires Paris Ouest (Hôpital Européen Georges Pompidou, Vaugirard, Corentin Celton) and Groupe Hospitalier Paris Saint Joseph. All patients aged 18 years or more, with a positive VZV PCR in cerebrospinal fluid (CSF) within the study period (January 2011 to December 2018), were included. VZV PCR was performed in the virology department of Cochin Hospital (Paris, France), along with systematic HSV PCR and more recently systematic enterovirus PCR.

### 2.2 Ethics

With the agreement of the heads of the concerned departments, medical files were de‐identified and consulted in each center. The study was approved in September 2018 by the Sud‐Méditerranée I Ethical Research Committee (number SI 18 09 03 66527) and by the French Data Protection Authority (CNIL number 2206174v0) responsible for ethical issues and protection of individual data collection. Patients received a letter informing them of the retrospective study in January 2019. After the retrospective data collection phase, patients were contacted by phone, mail, or email from February to May 2019 to confirm their consent, update their file, and collect follow‐up data. They could withdraw from the study at any time and ask for the detailed protocol, in accordance with French legislation.

### 2.3 Data collection

Clinical data were collected from the electronic medical records. The clinical presentation (signs, symptoms, date of onset, neurological symptoms with details on central or peripheral nervous system involvement) was inferred from the medical chart. Independence of the patients prior to the infection was assessed by the modified Rankin Scale (mRS) (Torres et al., 2020). Patients were considered as immunosuppressed or at risk depending on their comorbidities: mild risk included diabetes, pregnancy, HIV infection with CD4+ lymphocyte count > 500/mm^3^, and/or chronic renal disease whereas severe risk included malignancy (solid tumor or hematologic), HIV infection with CD4+ lymphocyte count < 500/mm^3^, transplant, and ongoing immunosuppressive treatments. Biological data collected included C‐reactive protein level (CRP), complete blood count and cerebrospinal fluid (CSF) features [protein and glucose levels, VZV viral load and interferon (IFN) alpha level]. Cerebral magnetic resonance imaging (MRI) and electroencephalograms (EEG) were also reviewed when available.

### 2.4 Definitions of neurologic complications

Among all patients with positive VZV PCR in the CSF, complications were defined as detailed in Table 1 (Gilden et al., 1994; Persson et al., 2009; Robillard et al., 1986; Steiner & Benninger, 2018; Venkatesan et al., 2013). Meningitis was defined by the association of a pleocytosis (≥5 nucleated cells/mm^3^ in the CSF) and suggestive symptoms of meningitis, with negative CSF bacterial cultures and without focal central or peripheral symptoms. After a careful review of medical charts, patients with alternative diagnoses not responding to the VZV neurological complications classification were excluded.

**TABLE 1:** Classification of neurological complications from VZV

	Meningitis *Persson 2009*	Pleocytosis ≥5 nucleated cells/mm^3^ (CSF) Signs and symptoms of meningitis No focal deficit, no coma, no alteration of mental status No peripheral involvement Negative bacterial CSF culture
CNS	Meningoencephalitis *Persson 2009* *Venkatesan 2013*	Motor or sensitive central focal deficit or seizures or alteration of mental status or abnormal electroencephalogram (EEG) or abnormal brain MRI suggestive of encephalitis
	Myelitis *Gilden 1994*	Motor or sensitive medullar deficit Positive medullar MRI (spinal cord T2 hypersignal)
	Stroke	Motor or sensitive central deficit Positive cranial MRI typical from neurologic stroke
PNS	Peripheral involvement *Robillard 1986* *Steiner 2018*	≥1 cranial nerve palsy or ≥1 peripheral radiculopathy *Isolated expected pain related to a zoster outbreak was not considered here*
Isolated viral detection		Positive VZV PCR in the CSF Not corresponding to any other neurologic classification

Abbreviations: CNS, central nervous system; CSF, cerebro spinal fluid; EEG, electroencephalogram; MRI, magnetic resonance imaging; PCR, polymerase chain reaction; PNS, peripheral nervous system; VZV, varicella zoster virus.

### 2.5 Medical care and follow‐up

Hospital stay data (location, duration, intensive care unit requirement), treatments (oral or intravenous antiviral therapy, dosage, delay, and duration of treatment), and discharge data were collected. At follow‐up contact, sequelae were assessed by the patient and the main investigator. The patient determined the recovery date (defined as the time of full recovery or when symptoms were stable). An unfavorable outcome was defined by an incomplete recovery (any persistent symptom or sequelae) and/or death related to VZV infection. A favorable outcome was defined by a full recovery (no sequelae, no persistent symptom in comparison to the status before infection). Level of disability at recovery date was assessed by the mRS. Change in mRS was defined by the difference from baseline mRS (mRS delta = mRS at recovery date – prior to infection mRS). The type of sequelae was specified by binary questions and free comments by the patients.

### 2.6 Statistical analysis

An analysis was conducted to identify clinical or biological prognostic factors that were associated with: a) unfavorable outcome (mortality and/or incomplete recovery) and b) increase of mRS delta. Descriptive statistics were obtained by reporting median and interquartile range (IQR) for continuous variables, while frequencies and proportions for categorical variables, as appropriate. When needed, the normality of the corresponding variables was assessed by the Shapiro‐Wilk test. Univariate analyses were conducted using Mann‐Whitney tests to compare non‐parametric data and Fischer or Chi‐squared tests to compare qualitative data, as appropriate. Multivariate analysis was performed using multiple logistic regression to assess the risk factors associated with unfavorable outcome. A secondary sensitivity analysis was conducted by excluding the deceased patients to assess the functional outcome (sequelae) in the survivors. A secondary analysis of risk factors for mortality was performed using logistic regression in the CNS group as all deaths occurred in this group. To assess the parameters associated with the mRS delta, Spearman correlation coefficients and Wilcoxon tests were calculated with a confidence interval of 95%. Multivariate analysis was performed using a Poisson regression model as mRS can be considered as count data. Analyses were conducted with R software (3.5.2). All tests were two‐sided, with a significance set at *p *< .05.

## 3 RESULTS

Between January 2011 and December 2018, 72 patients had a positive VZV PCR in the CSF and were included in this study. All patients had negative CSF bacterial culture and negative CSF HSV PCR; CSF enterovirus PCR was negative in all of the tested patients. Median age was 51 years (IQR [34‐75]), 38 patients (53%) were male, and 30 (42%) were considered immunosuppressed or at risk (11 mild, 19 severe). Before infection, 39 patients (54%) had neither symptoms nor limitation of their activities (mRS = 0), and median mRS for the whole group of patients was 0 [0–2]. Meningitis was diagnosed in 26 cases, central involvement (CNS group) in 27 cases with 22 meningoencephalitis, 5 myelitis and 3 strokes (3 CNS involvements were combined syndromes: 1 meningoencephalitis and stroke, 1 myelitis and stroke, 1 myelitis and meningoencephalitis), peripheral involvement (PNS group) in 16 cases (15 with cranial nerves involvement, 1 with motor neuropathy), and 3 patients had isolated replication of VZV in the CSF (positive PCR but no criteria for neurological complications from VZV). Patient's characteristics are described in Table 2.

**TABLE 2:** Characteristics of 69 patients with neurological VZV infection

	Meningitis (*n* = 26)	CNS (*n* = 27)	PNS (*n* = 16)
Age, years (median [IQR])	34 [24–48]	63 [52–81]	68 [37–82]
Female, *n* (%)	18 (70%)	9 (33%)	6 (38%)
Immunosuppressed or at risk, *n* (%)	6 (23%)	19 (70%)	4 (25%)
Severe immunosuppression, *n* (%)	4 (15%)	12 (44%)	3 (19%)
Anterior mRS (median [IQR])	0 [0–0.8]	2 [0–3]	0 [0–1]
Fever > 38°C, *n* (%)	19 (73%)	11 (41%)	4 (25%)
Herpes zoster, *n* (%)	17 (65%)	16 (59%)	12 (75%)
CSF protein level, g/L (median [IQR])	0.9 [0.7–1.3]	1 [0.6–2.1]	0.9 [0.7–0.9]
CSF VZV viral load, log (median [IQR])	5.2 [4.5–5.6]	4.7 [4.1–5.4]	4.3 [3–5.6]
CSF IFN, IU/L (median [IQR])	0 [0‐0] *n* = 21	0 [0‐3] *n* = 26	0 [0–0]
CRP, mg/L (median [IQR])	1.3 [0–3.7]	17.6 [8–45.8]	1.1 [0–5.3]
Antiviral treatment, *n* (%)	24 (92%)	26 (96%)	16 (100%)
Treatment duration, days (median [IQR])	10 [7–14]	21 [14–23]	14 [10–21]
**Outcomes (*n* = 62/69)**:			
Completed long term follow‐up, *n* (%)	25 (96%)	22 (81%)	15 (94%)
Unfavorable outcome, *n* (%)	6 (24%)	18 (82%)	13 (87%)
Deaths linked to VZV, *n* (%)	0 (0%)	8 (36%)	0 (0%)
Incomplete recovery, *n* (%)	6 (24%)	10 (45%)	13 (87%)
mRS delta (median [IQR])	0 [0–0]	1 [1–3]	1 [1–2]
Follow‐up, years (median [IQR])	3.4 [2–4.4]	0.5 [0.3–3.8]	2.2 [1.2–3.7]

Abbreviations: CNS, central nervous system; CRP, C‐reactive protein; CSF, cerebrospinal fluid; IFN, interferon; IQR, interquartiles 25–75; mRS, modified Rankin scale; PCR, polymerase chain reaction; PNS, peripheral nervous system; VZV, varicella zoster virus. Unfavorable outcome was defined as mortality or incomplete recovery (any persistent symptom or sequelae). Disability linked to the neurological infection was assessed by the mRS delta defined by the difference from baseline mRS (mRS delta = mRS at recovery − mRS prior to VZV infection).

### 3.1 Meningitis (*n* = 26)

The most frequently reported meningitis symptoms were headache (*n* = 26, 100%), neck stiffness (*n* = 19, 73%), and phonophotophobia (*n* = 16, 62%). Herpes zoster was present in 17 patients (65%), mostly in the thoracic dermatome (*n* = 9). Most patients (73%) had fever > 38°C. Median delay between onset of headache, zoster, fever, and the lumbar puncture were 3, 2, and 2 days, respectively. The median cell count in CSF was 153/mm^3^ [103–350], mostly lymphocytes (91% in median) but with possible presence of neutrophils (median 1%). CSF protein level was elevated (> 0.4 g/L) in 25 patients (36%); Eight patients (40%) had a CSF/plasma glucose ratio < 0.5. IFN alpha in the CSF was available in 21 patients and undetectable in 17 of them (81%). Median VZV CSF viral load was 5.2 log copy/ml. When performed, brain MRI was normal in 4/5 patients. The fifth patient had equivocal MRI findings (increased signal intensity), but he had no focal deficit on physical examination and these radiological abnormalities were not found on early control MRI. All but two patients (92%) received intravenous (IV) acyclovir at a median dose of 15 mg/kg/8 h [10–15]. Median duration of IV treatment was 7 days [4–10]. Median total treatment duration (oral + IV) was 10 days [7–14]. The median duration of hospital stay was 7 days [5–10]; no patient required intensive care management during hospitalization.

### 3.2 CNS group (*n* = 27)

Among the 22 patients with meningoencephalitis, 15 (68%) were immunosuppressed, median age was 72 years [55–86], and median mRS before VZV infection was +2. Intensive care was needed in 13 cases (59%) and hospitalization duration was 24 days in median [13–40]. All eight patients who died from VZV infection were patients with meningoencephalitis (and/or myelitis, stroke).

Among the five patients with myelitis, the median age was 40 years [32–54] and all patients were severely immunosuppressed (three with HIV infection and less than 500/mm^3^ CD4+ lymphocytes, one on immunosuppressant therapy, and one with leukemia on chemotherapy). All five had thoracic transverse myelitis that extended up to C3 for one of them. Only two of them had concomitant multimetameric HZ. One patient with myelitis and meningoencephalitis died during hospitalization.

Three patients presented with stroke: one with concomitant HZ (cervical, ipsilateral to the brain infarction) and the other two with concomitant fever (one with meningoencephalitis and the other with myelitis), suggestive of VZV vasculopathy.

When performed, brain MRI was pathological in 6/15 patients with encephalitis, with most patients showing hyperintensity on FLAIR MRI. All patients with myelitis had abnormal spinal imaging (hyperintensity on T2 MRI). Cerebral infarction was visible on MRI in all three stroke patients.

### 3.3 PNS group (*n* = 16)

Fifteen patients presented with an involvement of one or multiple cranial nerves (cranial nerve II (*n* = 1), V (*n* = 3), VI (*n* = 1), VII (*n* = 12), VIII (*n* = 4), IX (*n* = 3), X (*n* = 1) with six patients presenting with multiple cranial nerves involvement), while one patient had a unilateral L4 radiculopathy with an ipsilateral axonal sensitive polyneuropathy from L2 to L4. Among the PNS group, 12 out of these 16 patients had concomitant herpes zoster (9 with Ramsay‐Hunt syndrome, 1 thoracic, 1 facial and ophthalmic, 1 L2 territory). The 4 remaining patients presented with headaches and facial palsy (*n* = 3) or deficit of the sixth cranial nerve (*n* = 1).

### 3.4 Outcomes

Follow‐up was obtained from 62 patients (86%), death related to VZV occurred in 8 of them (13%) (Table 2) and the median follow‐up duration was 2.9 years (IQR [1.5–4.4]) for the 54 remaining patients. All deceased patients had CNS involvement (8 with meningoencephalitis with 1 also presenting with myelitis and 1 with stroke). In the CNS group, only mRS prior to VZV infection was significantly associated with mortality in univariate logistic regression (odd‐ratio 2.5 [1.2–7], *p* = .035), whereas age and immunosuppressed status were not (OR 1.01 [1–1.1] *p* = .533 and OR 1.2 [0.2–11] *p* = .857, respectively).

An unfavorable outcome was reported in 37 patients out of 62 (60%): 29 incomplete recoveries and 8 deaths. This rate was higher in the PNS group with 13/15 patients having at least 1 sequela (sleep or attention disorder, headache, depression, facial palsy, etc., compared to baseline status), but 80% of them recovered their prior independence. Six patients with meningitis reported an incomplete recovery (24%): attention disorder (*n* = 4), chronic headaches (*n* = 2), depression (*n* = 2), post herpetic neuralgia (*n* = 2), sleep disorder (*n* = 1), but 100% of them went back to work and to their daily activities. In the entire cohort, higher age (*p* < .001), higher mRS prior to infection (*p* < .001), altered mental status (*p* = .008), and characteristics of neurological involvement (CNS and PNS compared to meningitis, *p* < .001) were significantly associated with an unfavorable outcome. Multivariate logistic regression model confirmed the significant association of unfavorable outcome with a higher mRS prior to infection (OR 3.1 [1.4–8.8], *p* = .012) and the clinical group (compared to meningitis, PNS OR 22 [4–181], *p* = .001; CNS OR 6.2 [1.3–33], *p* = .03) (Table 3). These results were confirmed by a sensitivity analysis excluding the 8 deceased patients.

**TABLE 3:** Risk factors for unfavorable outcome in neurological complications from VZV

Risk factor	Favorable (*n* = 25)	Unfavorable (*n* = 37)	OR (univariate model)	OR (final multivariate model)
Age, years	32 [23–48]	68 [48–81]	1.1 (1.0–1.1, *p* < .001)	–
Prior‐to‐infection mRS	0 [0–0]	1 [0–3]	3.3 (1.7–8.1, *p* = .002)	3.1 (1.4–8.8, *p* = .012)
Immunosuppression, *n* (%)	7 (28)	17 (46)	2.2 (0.8–6.8, *p* = .158)	–
Fever, *n* (%)	17 (68)	14 (38)	0.3 (0.1–0.8, *p* = .022)	
Altered mental status, *n* (%)	4 (16)	18 (49)	5.0 (1.5–19.6, *p *= .012)	–
CRP, mg/L	1.4 [0–5.5]	5 [0.6–18.6]	1.1 (1.0–1.1, *p* = .079)	–
CSF protein level, g/L	0.9 [0.7–1.3]	0.9 [0.7–1.5]	1.2 (0.9–2.0, *p* = .305)	–
CSF IFN level, IU/L	0 [0–0])	0 [0–3]	1.0 (0.99–NA, *p* = .461)	–
Meningitis Group, *n* (%)	19 (76)	6 (16)	–	–
CNS Group, *n* (%)	4 (16)	18 (49)	14.3 (3.8–66.8, *p* < .001)	6.2 (1.3–33.3, *p* = .026)
PNS Group, *n* (%)	2 (8)	13 (35)	20.6 (4.2–159.1, *p* = .001)	21.6 (4.0–181.8, *p* = 0.001)

Abbreviations: CNS, central nervous system; CRP, C‐reactive protein; CSF, cerebrospinal fluid; IFN, interferon; IQR, interquartiles 25–75; mRS, modified Rankin scale; OR, odd‐ratio; PNS, peripheral nervous system; VZV, varicella zoster virus. Unfavorable outcome was defined as mortality or incomplete recovery (any persistent symptom or sequelae). Descriptive statistics were obtained by reporting median and interquartile range [IQR 25–75] for continuous variables, while frequencies and proportions for categorical variables, as appropriate.

Median mRS delta was +1 [0–2]. A multivariate Poisson regression model identified three risk factors associated with a higher mRS delta: age (coefficient 0.014, *p* = .0003), the clinical group (compared to meningitis, PNS 1.8 *p* < .0004, CNS 1.7 *p *< .0001), and the CSF protein level (0.15, *p* < .0001).

## 4 DISCUSSION

We report a large series of VZV neurological complications with positive CSF VZV PCR, describing 72 patients, with long‐term follow‐up available in 86% of cases. The observed neurological complications were heterogeneous and involved every level of the neurologic system from CNS to PNS with also milder forms of aseptic meningitis. The current study adds greatly in the effort to describe the clinical presentation of these types of patients and also to identify prognostic risk factors associated with a higher morbimortality.

With the routine measurement of VZV PCR in the CSF, more cases are being identified and VZV neurological complications are increasingly being reported. For example, VZV is now considered among the three first causes (along with enterovirus and HSV) of viral meningitis (Kupila et al., 2006). More data about outcome and prognosis are needed to guide therapeutic strategies, especially for VZV aseptic meningitis. Indeed, these forms are strongly felt to be milder than CNS involvements, but, to date, no study gives sufficient reassuring information to lighten their treatment compared to meningoencephalitis therapeutic recommendations (Stahl et al., 2017). A major limitation of our knowledge of aseptic meningitis outcomes is the paucity of data specifically assessing it; previously published studies with similar designs reported on up to 34 patients (Aberle et al., 2005; Becerra et al., 2013; Corral et al., 2020; De La Blanchardiere et al., 2000; Haanpaa et al., 1998; Ihekwaba et al., 2008; Kaewpoowat et al., 2016; Pahud et al., 2011; Persson et al., 2009; Skripuletz et al., 2018; Tabaja et al., 2020), but follow‐up data were available for only 12 patients at 6 months (Corral et al., 2020; Persson et al., 2009). Other studies have included meningoradiculitis with meningitis cases (Aberle et al., 2005; Echevarria et al., 1994; Kim et al., 2017), which in our opinion, reflects a different pathophysiological process leading to different sequelae. To date, there is also poor evidence about the role of immunosuppression in the outcome of VZV neurological complications.

Our most notable finding was that patients with meningitis had a favorable outcome (in terms of mortality and sequelae) compared to patients with complications involving the PNS or CNS (of note, 24 out of 26 patients with meningitis received antiviral treatment). Most patients with meningitis were young, had no significant past medical history, and had a typical aseptic meningitis presentation (fever, headache, and meningeal signs). HZ was present in two‐thirds of patients, but as previously described, its absence did not rule out the diagnosis of VZV meningitis. Inflammatory syndrome was low, and the most frequent CSF abnormality was a lymphocytic pleiocytosis. Presence of polynuclear neutrophils or low glucose in the CSF did not rule out the diagnosis. Thus, an aseptic meningitis with hypoglycorrhachia could evoke a VZV infection as described in 40% of the patients in our cohort. In the meningitis group, no patient died from VZV infection and only six (24%) reported an incomplete recovery which was significantly lower than in the CNS and PNS groups. In contrast, members of the CNS group had poorer outcomes than previously described in published cohorts (Corral et al., 2020; Grahn & Studahl, 2015) with a higher mortality (36%) and a high rate of sequelae (45%). Of note, in our study, the overweight of female gender in the meningitis group (70% versus 33% and 38% for CNS and PNS groups, respectively) and the younger age (median age 34 versus 63 and 68 respectively) could be effects behind the improved outcome.

Another strength of our study is that we only included patients with PCR‐confirmed VZV infection in the CSF and no other pathogen documented, in contrast to previous works which included heterogeneous populations like the work by Corral et al. (2020), who studied 98 patients with VZV neurological complications with only 27 positive VZV PCR in the CSF out of the 46 available results. These stringent criteria allowed us to specifically dissect the neurological complications linked to VZV only, and not to potential other agents. However, these two studies are highly complementary, as ours describes cases requiring lumbar puncture (therefore self‐selecting for more CNS involvement) whereas the Corral study likely describes milder cases with more PNS involvement. A recent study by Herlin et al. (2021) described the clinical presentation, outcome, and prognostic factors of 92 patients with VZV encephalitis having positive VZV PCR in the CSF or intrathecal anti‐VZV IgG. As the latter are only rarely used in practice in France, they were not part of our inclusion criteria or data collection, which may constitute a selection bias.

Follow‐up data permitted us to assess two well‐defined outcomes (full recovery and mRS delta) and to conduct a multivariate analysis that identified risk factors associated with a poorer prognosis: higher age, higher prior‐to‐infection mRS, CNS or PNS involvement compared to aseptic meningitis, and CSF protein level. Older age is a well‐known risk factor for VZV reactivation and complications (Kaewpoowat et al., 2016; Nagel & Gilden, 2014; Schmader, 2001). Elevated CSF protein level had not been previously described as associated with a poorer prognosis. This could reflect the alteration of the blood brain barrier by the viral infection (or the intensity of the inflammation associated with the viral infection and/or immune response). Viral load was not associated with prognosis, which is in accordance with one (Rottenstreich et al., 2014), out of two studies (Persson et al., 2009) that assessed this parameter. Interestingly, immunosuppression was not associated with mortality or incomplete recovery, as reported in another recent study (Corral et al., 2020).

Our study was limited by the potential for misclassification bias. Patients in our cohort were not classified in groups according to file coding, but rather, after careful study of medical records. When altered mental status (AMS) was reported, patients were automatically classified in the CNS group. Eight patients with AMS also had other potential causes of confusion: 5 were ≥77 years‐old, 1 had acute urinary retention, 1 acute renal failure, and 1 was on antipsychotic treatment. None of them further developed clinical or radiological sign of encephalitis. Such patients could initially have been diagnosed with meningitis as we know that these entities overlap. These patients could have a severe meningitis with AMS or a meningoencephalitis without obvious encephalitis at the time of the lumbar puncture. Our results showed that AMS was associated with a poorer outcome in univariate analysis, but this effect was no longer significant in the multivariate model after adjusting for the clinical group. Being in the CNS group was associated with poorer outcome. This adds to the point that patients with initial AMS and no other signs of encephalitis might be more severe than “simple” aseptic meningitis even if AMS was their only CNS sign. The other biases related to our study design were minimized by 1) the inclusion of all patients with positive CSF VZV PCR during the study period in the participating centers, limiting recruitment bias; 2) the stringent judgment criterion—full recovery is subjective but clinically relevant and subject to overestimation of sequelae rather than underestimation; and 3) updated follow‐up data by direct patient contact. This study was not designed to investigate VZV vasculopathy, as VZV PCR in the CSF is not routinely prescribed through stroke management and can be negative in such cases. Nevertheless, our follow‐up could have detected the occurrence of a new neurological deficit, but none of our patients’ evolution suggested the presence of VZV vasculopathy.

Our study allowed us to examine therapeutic practices in French hospitals, especially the therapeutic management of VZV aseptic meningitis. Physicians tend to treat these patients and prefer the IV antiviral therapy at high dose for at least 1 week. In the absence of guidelines on this matter in the medical literature, physicians seem to follow the management guidelines for VZV encephalitis instead. Considering the design of the study, we did not test the association between therapeutic regimen and outcome. Given the favorable outcome of VZV meningitis, a prolonged high dose IV treatment may not be necessary for young patients with no significant medical history and rapid improvement. Indeed, IV antiviral therapy induces longer hospital stays, greater risk of line‐related infection, and can lead to acute renal insufficiency. In our study, only two patients did not receive any antiviral therapy. They presented with typical aseptic meningitis and were discharged after symptomatic treatment. At follow‐up, they reported a full recovery. Although definite conclusions cannot be drawn from those isolated cases, this emphasizes the need for a prospective randomized trial in order to provide guidelines for the treatment of VZV meningitis specifically. Patients’ selection could be guided by the risk factors of poorer prognosis that we identified. Patients with older age or higher CSF protein level should not be included because they might need more aggressive treatment. Given the relative rarity of VZV meningitis, another interesting project might be to open a prospective registry that would capture VZV neurological complications and management.

## 5 CONCLUSION

In this retrospective study, neurological complications from VZV were diverse with heterogeneous outcomes: aseptic meningitis without other neurological complications had a good prognosis whereas CNS involvements presented a higher risk of mortality and PNS involved a higher risk of functional sequelae. Age, higher prior‐to‐infection Rankin score, and higher CSF protein level were risk factors of poorer prognosis. While these data need to be confirmed by prospective studies, they provide key information to guide future registries or controlled trials.

## CONFLICT OF INTEREST

The authors declare that they have no conflict of interest.

## AUTHOR CONTRIBUTIONS

All authors contributed to the study conception and design. Material preparation, data collection, and analysis were performed by Tiphaine Lenfant, Anne‐Sophie L'Honneur, Benoit Pilmis, and Adrien Michon. Statistical analyses were performed by Tiphaine Lenfant and Brigitte Ranque. The first draft of the manuscript was written by Tiphaine Lenfant and all authors commented on the previous versions of the manuscript. All authors read and approved the final manuscript.

### PEER REVIEW

The peer review history for this article is available at https://publons.com/publon/10.1002/brb3.2455.

## Data Availability

The datasets generated during and/or analyzed during the current study are available from the corresponding author on reasonable request.
